# Comparative evaluation of handwriting recognition by large language models (LLMs) in interpreting handwritten medication lists

**DOI:** 10.1016/j.rcsop.2026.100807

**Published:** 2026-05-27

**Authors:** Tom G. Jacobs, Laize Sílvia dos Anjos Botas Beca, Paul D. van der Linden, Merel van Nuland

**Affiliations:** a Department of Pharmacy, Tergooi Medical Center, Hilversum, the Netherlands; b Lurio University, Pharmacy Department, Nampula, Mozambique; c Julius Center for Health Sciences and Primary Care, University Medical Center Utrecht, Utrecht, the Netherlands.

**Keywords:** Artificial intelligence, Pharmacy, Large language model, Medication list, Digitization

## Abstract

**Introduction:**

Handwritten bedside medication lists remain common in healthcare, especially in low-resource countries, presenting challenges for digitization and automated safety checks. The introduction of large-language models may present an opportunity to facilitate digitization of handwritten medication lists.

**Objective:**

This study evaluated the accuracy of three GPT-based language models in recognizing handwritten medication lists, presented in Dutch.

**Methods:**

Thirty-three participants transcribed a list of 10 medications with dosage and administration instructions. These lists were processed by each model and scored for correct medication name, dosage, frequency, and route. The effect of writer characteristics on LLM performance was assessed using the Mann-Whitney *U* test (sex, handedness, ink colour) and Kruskal-Wallis H-test (handwriting style: cursive, print, mixed).

**Results:**

GPT 4.1 achieved the highest accuracy, followed by GPT4o, both outperforming GPT4o-mini (*p* < 0.001). Recognition strongly correlated with human legibility (ρ = 0.655; p < 0.001). Print handwriting and blue ink resulted in higher recognition than cursive or mixed styles and black ink. Complex dosing instructions were most error-prone.

**Conclusion:**

GPT-based optical character recognition showed potential for scalable digitization of handwritten prescriptions, however, human oversight remains essential to ensure medication safety. Future research should validate performance in real-world, multilingual settings.

## Introduction

The global digital revolution in healthcare is advancing rapidly. However, in many countries, progress remains slow due to fragile health systems, limited resources, and high patient loads.^1^ In these settings, handwritten bedside medication lists are common and are often the primary source of medication information. Misinterpretation of these lists can cause medication errors, leading to patient harm, increased costs, and compromised medication safety.^2^ Digital health and artificial intelligence (AI) present opportunities to improve the accessibility and efficiency of healthcare services.^3^ Hence, the World Health Organization (WHO) has developed a Global Strategy on Digital Health, highlighting the role of digital health in creating cost-effective and efficient health systems to improve health safety.^4^

A major challenge in this transition is accurately capturing data from medical documents, especially since handwritten medication lists at the patient's bedside are still commonly used. Errors at this analog-to-digital step could cause inappropriate automated safety checks.^5^ However, converting these lists into digital form creates opportunities for further processing, such as automatically detecting drug–drug interactions (DDIs) instead of manual review. Optical character recognition (OCR) using AI to recognize patterns in text is a promising method for rapid digitizing of handwritten medication lists. Compared to rule-based systems, OCR generally performs better because it applies machine learning methods.^6^ Studies show that OCR can digitize medication prescriptions with high accuracy (70–98%).^7^, ^8^, ^9^ These studies use specialized OCR systems rather than large language models (LLMs), which are designed for broader pattern recognition. As LLMs improve, integrating OCR capabilities into these models, such as OpenAI's ChatGPT, may provide advantages over task-specific OCR systems. In this study, the aim is to assess the accuracy of different versions of ChatGPT for evaluation of handwriting recognition in digitizing handwritten medication lists.

## Methods

### Participants and procedure

Pharmacy technicians and pharmacists from a single regional hospital (Tergooi Medical Center, Hilversum, The Netherlands) were invited to voluntarily participate in this study through direct in-person recruitment by the investigators during routine working hours between June and July 2025. Prior to participation, all participants were instructed to transcribe the medication lists exactly as they would normally write in daily clinical practice. Other than using the participant's own pen, no specific handwriting instructions, time limits or formatting requirements were provided to obtain handwriting samples representative of routine clinical documentation.

Participants were randomly assigned to complete either List 1 or List 2 from Supplementary Table 1. Each list contained 10 medication names with dosages and instructions. The medication lists were in Dutch; the participants' primary language. Demographic information (sex and handedness), ink colour and writing material were recorded, and participants manually transcribed the medication list. The completed forms were photographed by the investigators. A prompt was developed to enable the GPT model to process the handwriting and identify the medications. The prompt and photos were processed by three different OpenAI GPT models: GPT-o4, GPT-o4-mini and GPT-4.1.

### Data analysis

Digitized medication lists were compared with the original lists to calculate the percentage of correct identifications and errors. Scoring was based on correct drug name, dosage, frequency, and route (maximum score = 4). Errors in drug names were further categorized as (1) character-level errors (e.g., spelling or writing mistakes; 0.5 points) or (2) word-level errors (0 points), in which the drug name was substituted by another. The total score for each list was calculated by summing the individual drug scores per GPT model (maximum total score: 40).

Additional data collected from the handwritten lists included handwriting style (cursive, print or mixed), scored by the investigators after reaching consensus. Human legibility was assessed by a panel of 10 participants using a 5-point Likert scale (1 = poor readability, 5 = excellent). The mean score was used for further analysis.

### Statistical analysis

Analyses were performed using R (version 4.4.2) and SPSS (version 28). To compare the overall performance of the three GPT models, a Friedman test was conducted, as the data were non-normally distributed. Post-hoc pairwise comparisons were conducted with the Wilcoxon Signed Ranks test. Spearman's rank correlation was used to assess the relationship between mean human legibility scores (range 0–5) and GPT model performance (range 0–40).

The effect of writer characteristics on LLM performance was studied using the best-performing GPT model. For binary variables (sex, handedness, ink colour), the Mann-Whitney *U* test was applied, while the Kruskal-Wallis H-test was used for the categorical variable of handwriting style (cursive, print, mixed).

Descriptive statistics were used to report the medication name accuracy and whether more complex dosing descriptions (e.g., no strength/dosage reported, ‘as needed’ dosing, or time-specific dosing) were more prone to misinterpretation.

## Results

Thirty-three participants completed either medication list 1 (*n* = 17) or list 2 (*n* = 16) of which 17 were pharmacists and 16 were pharmacy technicians. Participant demographics are presented in Table 1. The three models differed significantly in handwriting recognition performance (Friedman test: X^2^(2) = 52.8, *p* < 0.001). Median (standard deviation (SD)) accuracies were as follows: GPT4o-mini (34.0; 4.50), GPT4o (38.0; 3.83), and GPT 4.1 (38.5; 3.26). Post-hoc pairwise comparisons showed that both GPT4o and GPT 4.1 significantly outperformed GPT4o-mini (*p* < 0.001). However, no significant difference was observed between GPT4o and GPT 4.1 (*p* = 0.062).

**Table 1:** Participant demographics.

Demographic		Number (percentage) (total *n* = 33)
Sex	Female	25 (76%)
	Male	8 (24%)
Handedness	Right	26 (79%)
	Left	7 (21%)
Writing material	Pen	33 (100%)
Ink colour	Blue	26 (79%)
	Black	7 (21%)
Handwriting style	Cursive	4 (12%)
	Print	19 (58%)
	Mixed	10 (30%

Human legibility of the handwritten medication lists was significantly associated with GPT recognition performance. Spearman's rank correlation showed positive correlations between human legibility scores and recognition performance across all three models: GPT4o-mini (ρ = 0.667, *p* < 0.001), GPT4o (ρ = 0.625, p < 0.001), and GPT 4.1 (ρ = 0.655, p < 0.001).

Handwriting style had a significant effect on GPT 4.1 recognition performance (Kruskal-Wallis test: X^2^(2) = 10.42, *p* = 0.005). Post-hoc analyses showed that print outperformed both cursive (*p* = 0.044) and mixed styles (*p* = 0.024), whereas no difference was found between cursive and mixed styles (*p* = 1.000). Ink colour also influenced GPT 4.1 performance, with blue ink outperforming black ink (Mann-Whitney U: *p* = 0.039). Neither sex (*p* = 0.476) nor handedness (*p* = 0.415) was associated with GPT 4.1 handwriting recognition performance.

Overall performance of GPT 4.1 for handwritten recognition of medication names was high. Only three medications – ciprofloxacin (2/17), Microgynon® (2/17), and carbamazepine (2/16) – were misread (word-level error) in two of the handwritten medication lists. For other medications, word-level errors were observed in one or zero lists.

Complex dosing instructions were more frequently misinterpreted by GPT 4.1. Dosing and frequency information was misread in 14% of entries when no strength or dosage information was reported, 35% of entries with “as needed” instructions, and 27% of entries that included specific timing instructions. In contrast, for medications without additional dosing complexity, only 3% of dosing and frequency fields were misread.

## Discussion

This study examined the performance of three GPT-based language models in digitizing handwritten medication lists, with the goal of assessing their potential to support digital transformation in healthcare systems where paper prescribing remains common. This is the first known study to assess the performance of LLMs in digitizing handwritten medication lists. Overall, GPT4o and GPT 4.1 demonstrated high accuracy in recognizing handwritten medication names, aligning with previous findings that AI-enabled OCR can effectively extract data from medical documents.^6^, ^7^, ^8^, ^9^ However, none of the previous studies used a widely available LLM for OCR.^6^, ^7^, ^8^, ^9^ The results from this study indicate that LLM-integrated OCR may offer a scalable solution for rapid digitization of bedside medication lists. Consequently, enhanced digitization can support secondary applications such as automated DDI checking and clinical decision support.

A clear performance gradient was observed: GPT 4.1 achieved the highest accuracy, followed by GPT4o, both outperforming GPT4o-mini. These findings suggest that advances in model and multimodal architecture enhance handwriting recognition capabilities. Similar improvements in GPT models have also been reported in medical education and clinical decision support.^10^, ^11^ The strong association between human-perceived legibility and model performance indicates that LLM-based handwriting recognition mirrors human reading constraints.^11^, ^12^ Clear handwriting remains a critical patient safety measure, and improvements in documentation practices could directly enhance the accuracy of AI-assisted digitization. Print handwriting yielded better accuracy than cursive or mixed styles, and blue ink was more accurately recognized than black ink, likely due to greater visual contrast on white paper.

Despite strong performance for medication names, accuracy declined for complex dosage instructions, such as conditional or time-specific regimens. Errors in these fields may impair detection of inappropriate drug use or DDIs. Additionally, because generative models aim for internal consistency, misinterpretation of one element (e.g., dosage) may lead to substitution of another. For example, ciprofloxacin 500 mg was occasionally misread as 30 mg. As this formulation does not exist, the model replaced the drug with gliclazide. Although infrequent, such word-level substitutions pose a notable patient safety risk and highlight the need for human verification before clinical use. This behaviour illustrates a key characteristic of generative models: their tendency to prioritize coherent output over literal transcription accuracy^13^; which, while advantageous in many natural language tasks, introduces critical safety considerations in this setting.^14^

This study has limitations. The handwritten lists were in Dutch, whereas the primary languages in many low- and middle-income countries differ; however, performance in widely represented languages such as English, Portuguese, or Spanish may be equal or superior. Additionally, real-world clinical handwriting may be more variable and less legible than the controlled samples used here. On the other hand, bedside medication lists should inherently maintain high readability to ensure patient safety. Therefore, this limitation is not expected to substantially affect the applicability in clinical practice.

## Conclusion

In conclusion, handwritten medication lists are fast and flexible but often unreliable due to poor legibility and inconsistent formats, limiting their use for safety checks and data analysis. GPT-based handwriting recognition shows promising potential for automated medication list digitization, but human oversight remains essential to ensure patient safety. Future research should focus on validating handwriting recognition tools in real-world clinical environments, including diverse languages, clinical specialties, and levels of documentation quality.

## CRediT authorship contribution statement

**Tom G. Jacobs:** Writing – original draft, Project administration, Methodology, Investigation, Formal analysis, Data curation, Conceptualization. **Laize Sílvia dos Anjos Botas Beca:** Writing – review & editing, Investigation. **Paul D. van der Linden:** Writing – review & editing, Supervision, Methodology, Investigation, Conceptualization. **Merel van Nuland:** Writing – original draft, Supervision, Methodology, Investigation, Formal analysis, Data curation, Conceptualization.

## Declaration of competing interest

The authors declare that they have no known competing financial interests or personal relationships that could have appeared to influence the work reported in this paper.
